# Integrated Computational Analysis Reveals Structurally Destabilizing Missense Variants in the *PDX1* Transcription Factor

**DOI:** 10.3390/genes17030273

**Published:** 2026-02-27

**Authors:** Elsadig Mohamed Ahmed

**Affiliations:** Department of Medical Laboratory Sciences, College of Applied Medical Sciences, University of Bisha, P.O. Box 551, Bisha 61922, Saudi Arabia; emfadlalla@ub.edu.sa

**Keywords:** PDX1 transcription factor, missense variant prioritization, protein structural destabilization, monogenic diabetes (MODY4), molecular dynamics simulation, variant pathogenicity prediction, β-cell transcriptional regulation

## Abstract

Background/Objective: Pancreatic and duodenal homeobox 1 (PDX1) is a key transcription factor required for pancreatic development and maintenance of β-cell function. Genetic variants in PDX1 have been associated with monogenic forms of diabetes, including maturity-onset diabetes of the young type 4 (MODY4). However, the func-tional consequences of many reported non-synonymous single-nucleotide polymorphisms (nsSNPs) in PDX1 remain unclear. In this study, an integrated in silico approach was applied to systematically identify and characterize po-tentially deleterious nsSNPs in the PDX1 gene. Methods: Missense variants were retrieved from public databases and evaluated using multiple sequence- and structure-based prediction tools to assess functional impact, disease association, protein stability, and structural consequences. Variants considered deleterious were further examined through three-dimensional structural modeling and molecular dynamics simulation. Results: Several nsSNPs were identified with consistent predictions of pathogenicity, reduced protein stability, and pronounced structural and dynamic perturbations. Variants including R197G, Y170N, and T151K in the PDX1 Protein were considered the highest deleterious mutants. Conclusion: These findings will provide insight into the molecular mechanisms by which PDX1 mutations may contribute to β-cell dysfunction and diabetes development and offer a rational framework for prior-itizing variants for experimental validation and clinical interpretation.

## 1. Introduction

Diabetes mellitus (DM) comprises a heterogeneous group of metabolic disorders characterized by chronic hyperglycemia arising from insufficient insulin secretion, impaired insulin action, or both. Insulin is synthesized and released by pancreatic β-cells within the islets of Langerhans and is essential for maintaining glucose homeostasis. Dysregulation of insulin production or signaling leads to sustained elevations in blood glucose levels and underlies the development of diabetes and its associated complications [1,2,3,4,5,6].

Among the various forms of diabetes, type 1 and type 2 diabetes mellitus are the most prevalent. However, monogenic diabetes represents a distinct and clinically important category caused by pathogenic variants in single genes that directly affect pancreatic β-cell development, function, or survival [3,7]. These monogenic forms differ substantially from polygenic diabetes in their genetic basis, clinical presentation, and therapeutic management [3,7,8].

Maturity-onset diabetes of the young (MODY) is a well-recognized subtype of monogenic diabetes inherited in an autosomal dominant manner and typically manifests during adolescence or early adulthood, often before the age of 25 [3,7]. MODY is caused by mutations in genes that regulate key processes such as β-cell function, insulin biosynthesis, glucose sensing, and transcriptional control of pancreatic genes [7,8,9]. Although MODY is relatively rare, accurate genetic diagnosis is essential because its clinical features may overlap with type 1 or type 2 diabetes, potentially leading to misclassification and suboptimal treatment [3,9].

Multiple MODY subtypes have been identified, each associated with mutations in distinct genes critical for β-cell biology. Among these, MODY type 4 (MODY4) is caused by mutations in the pancreatic and duodenal homeobox 1 (PDX1) gene [5,8,10]. This PDX1 gene, also known as insulin promoter factor 1 (IPF1), encodes a homeodomain-containing transcription factor that plays a central role in pancreatic organogenesis and maturation of β-cells [11,12].

Located on chromosome 13q12.1, the human PDX1 gene encodes a 283–amino acid protein that regulates the transcription of several genes essential for pancreatic function, including insulin, glucokinase, and somatostatin [2,9]. During embryonic development, PDX1 is indispensable for pancreatic formation, while in adult β-cells it is required for insulin gene transcription and glucose-responsive insulin secretion [5,8].

Pathogenic variants in PDX1 give rise to a wide spectrum of clinical phenotypes depending on mutation type and zygosity. Homozygous or compound heterozygous mutations can result in pancreatic agenesis and neonatal diabetes, whereas heterozygous mutations typically cause MODY4, characterized by progressive β-cell function impairment and reduced insulin secretion [13,14,15]. These observations underscore the exceptional sensitivity of PDX1 function to alterations in its protein structure.

Among genetic variants, non-synonymous single-nucleotide polymorphisms (nsSNPs) are of particular importance because they introduce amino acid substitutions that may alter protein folding, stability, DNA-binding affinity, or transcriptional activity [16,17]. In the case of transcription factors such as PDX1, even subtle amino acid changes within functional domains can disrupt regulatory interactions and lead to impaired β-cell gene expression [18,19].

Several PDX1 nsSNPs have been experimentally validated as pathogenic and shown to compromise transcriptional activity, nuclear localization, or protein stability, thereby contributing to β-cell dysfunction and diabetes development [8,10]. However, many reported PDX1 variants remain poorly characterized, and their functional consequences are unknown. Experimental validation of each variant is costly, effective, and time-consuming. This highlights the need for effective computational strategies to prioritize potentially deleterious mutations.

Advances in bioinformatics have led to the development of numerous tools capable of predicting functional, structural, and pathogenic effects of amino acid substitutions [20,21,22,23]. Integrating these multiple predictive approaches improves confidence in variant classification and enables identification of deleterious nsSNPs that necessitate further experimental validation. Such integrated computational pipelines have been successfully applied to pathogenic PDX1 variants and have provided valuable insights into genotype–phenotype relationships [19].

Functionally, pathogenic PDX1 variants can impair DNA binding, disrupt transcriptional regulation, or interfere with nuclear localization, ultimately reducing insulin gene expression and compromising β-cell function [15,24]. From a clinical perspective, accurate identification of pathogenic PDX1 mutations is essential for distinguishing MODY4 from other diabetes subtypes, thereby enabling personalized treatment strategies, including the use of oral hypoglycemic agents instead of insulin therapy when appropriate [25].

Considering the essential function of PDX1 in pancreatic biology and the expanding spectrum of reported missense variants, a systematic and integrative characterization of deleterious nsSNPs is essential. Therefore, the present study employed an integrated in silico framework to screen, prioritize, and characterize nsSNPs in the PDX1 gene. By combining functional prediction, pathogenicity analysis, protein stability assessment, structural modeling, molecular dynamics simulations, and three-dimensional visualization. This work aims to elucidate the structural and functional consequences of deleterious PDX1 variants and provide a rational basis for their prioritization in future experimental and clinical studies. Unlike previous studies that relied primarily on sequence-based pathogenicity prediction, this work integrates multi-tool consensus filtering with structural-dynamic analysis through molecular dynamics simulations, thereby enabling mechanistic evaluation of variant-induced structural and conformational alterations within the PDX1 homeodomain.

## 2. Materials and Methods

### 2.1. Retrieval of PDX1 Gene and Protein Information

The PDX1 gene and its corresponding protein sequence were retrieved from the National Center for Biotechnology Information (NCBI) and UniProt databases. The reference protein sequence in FASTA format (283 amino acids) was used as the wild-type template for all subsequent analyses. Structural and functional annotations, including domain organization and residue numbering, were verified to ensure consistency across prediction tools. This computational analysis is consistent with previously published research [19,26].

### 2.2. Identification of Non-Synonymous Single-Nucleotide Polymorphisms

The nsSNPs associated with the PDX1 gene were collected from the dbSNP database. Only missense variants resulting in amino acid substitutions within the coding region were selected for further evaluation (websites were accessed on 2 July 2025). Variants lacking protein-level annotation or mapping ambiguity were excluded. Minor allele frequency (MAF) information, when available, was recorded to distinguish rare from common variants.

### 2.3. Functional Impact Prediction of nsSNPs

To assess the potential functional consequences of PDX1 nsSNPs, multiple sequence-based prediction tools were employed. SIFT (accessed on 18 July 2025) was used to evaluate that amino acid substitutions affect protein function based on sequence homology and evolutionary conservation [27]. PolyPhen-2 (accessed on 20 July 2025) was applied to predict the impact of substitutions on protein structure and function using physical and comparative considerations [28]. FATHMM and PROVEAN (accessed on 22 and 24 July 2025, respectively) were additionally used to strengthen prediction confidence [29,30]. Variants that are predicted as deleterious by these multiple tools will be prioritized for downstream analysis.

### 2.4. Disease Association Analysis

The association of nsSNPs with disease phenotypes was evaluated using SNPs & GO and PhD-SNP servers (accessed on 26 and 28 July 2025, respectively). These tools integrate sequence features, evolutionary information, and functional annotations to classify variants as disease-related or neutral. The Reliability Index (RI) provided by both tools was used to assess the confidence level of each prediction. Variants with higher RI values, indicating greater prediction reliability [31,32], will be prioritized for further analysis.

### 2.5. Prediction of Protein Stability Changes

The effect of selected nsSNPs on protein stability was examined using I-Mutant and MuPro servers (accessed on 30 July and 2 August 2025, respectively). These tools are used to predict changes upon amino acid substitution by estimating the direction and magnitude of free energy change (ΔΔG) [33,34]. Variants that are predicted to significantly decrease the protein stability will be considered structurally disruptive. To further evaluate the stability alterations and the local conformational effects, the DynaMut2 tool (accessed on 8 August 2025) was used to analyze changes in protein flexibility and vibrational entropy. Variants with scores ≤ −2.5 will be considered protein destabilizing [35] and then will be prioritized for the second step of analysis.

### 2.6. Structural and Functional Consequence Analysis

MutPred2 (accessed on 12 August 2025) was employed to investigate potential molecular mechanisms affected by the prioritized nsSNPs, including alterations in secondary structure, post-translational modification sites, and functional motifs. Variants with a score > 0.5 are predicted to be pathogenic [36,37]. The HOPE server (accessed on 28 August 2025) was used to generate detailed structural interpretations of amino acid substitutions by analyzing differences in size, charge, hydrophobicity, and residue conservation within the three-dimensional context of the protein [38]. Variants identified as most deleterious using the HOPE tool will be prioritized for downstream analysis.

### 2.7. Protein Structure Modeling and Visualization

The three-dimensional structure of the wild-type PDX1 protein was generated using homology modeling approaches, with I-TASSER serving as the primary modeling platform (accessed on 2 November 2025). Mutant structures were constructed by introducing selected amino acid substitutions into the wild-type model [39,40,41]. Structural visualization and comparative analysis of wild-type and mutant proteins were performed using PyMOL v.2 (accessed on 12 January 2026) to assess changes in folding, secondary structure elements, and spatial orientation of key residues [42].

### 2.8. Molecular Dynamics Simulation

MD simulations were performed using GROMACS v2020.6 on Google Collaboratory Pro. The MD simulations were conducted to investigate the dynamic behavior and structural stability of the wild-type PDX1 protein and the selected mutant variants. Variants will be selected based on predefined quantitative thresholds, including ≥3/4 deleterious predictions across functional tools, |ΔΔG| ≥ 1 kcal/mol destabilization, localization within conserved homeodomain regions, predicted ligand-binding residues, high evolutionary conservation, and the DynaMut2 tool criteria (|ΔΔG| ≤ −2.5 kcal/mol destabilization). Variants ranked as most deleterious under these combined criteria were therefore prioritized for MD simulations analysis. The following detailed protocol was applied.

#### 2.8.1. Simulation Setup

In the GROMACS system, the OPLS-AA force field was applied for protein parameterization. The system was solvated using the TIP3P explicit water model. The protein was placed in a cubic simulation box with a minimum distance of 10 Å from the box edge. The system was neutralized with Na^+^ and Cl^−^ ions, and the ionic strength was adjusted to 0.15 M.

#### 2.8.2. Energy Minimization and Equilibration

Energy minimization was carried out using the steepest descent algorithm until the maximum force was below 1000 kJ/mol/nm. Equilibration consisted of a 100 ps NVT ensemble at 300 K using a V-rescale thermostat and a 100 ps NPT ensemble at 1 bar using a Parrinello–Rahman barostat. Heavy atoms were position-restrained during equilibration.

#### 2.8.3. Production Run and Analysis

A 100 ns production simulation was performed using a 2 fs integration time step under periodic boundary conditions. Electrostatic interactions were computed using PME with 1.0 nm cutoffs for both Coulombic and van der Waals forces. Structural analyses included RMSD, RMSF, Rg, SASA, hydrogen bond formation, and secondary structure content [43]. Comparative analyses were based on averaged mean ± SD values.

#### 2.8.4. Secondary Structure Analysis

Secondary structure content of the *PDX1* gene wild-type and mutants was quantified using DSSP analysis, and statistical values were calculated by averaging the number of secondary structural elements over the final 20 ns of each trajectory to ensure analysis of equilibrated conformations [44].

### 2.9. Data Integration and Variant Prioritization

**Figure 1:**
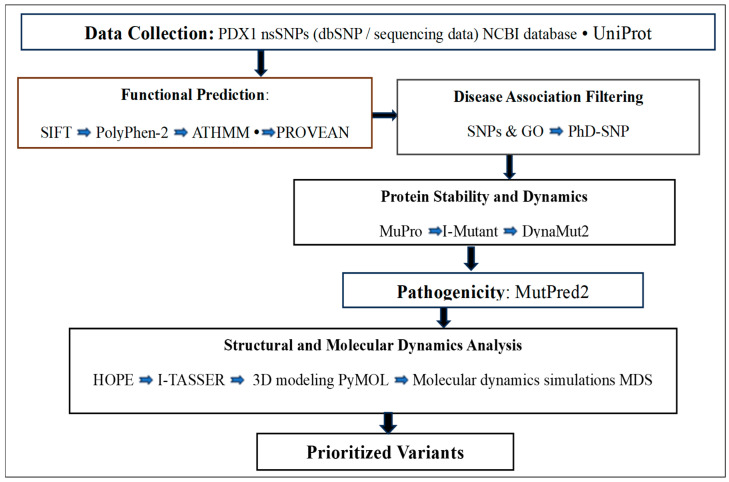
Flowchart outlining the identification and categorization of nsSNPs in the *PDX1* gene, with each step indicating the tools used. If an nsSNP is classified as deleterious by a particular tool at any step, it progresses to the next tool or step for further analysis.

The obtained results were systematically integrated to identify deleterious nsSNPs with the consistent prediction tools. Variants showing functional impact, pathogenicity, protein destabilization, and pronounced structural perturbations undergo detailed discussion. The overall workflow applied to identify and classify the potentially functional nsSNPs in the *PDX1* gene is illustrated in Figure 1.

### 2.10. Integrated Interpretation

To ensure interpretative consistency, effect size metrics from all computational tools were standardized prior to integration. ΔΔG values were uniformly interpreted such that negative values indicate destabilization and positive values indicate stabilization. Tool-specific pathogenicity outputs were mapped into a harmonized classification framework to reduce variability across predictive approaches. Structural alterations were interpreted strictly in relation to predicted stability changes and plausible functional consequences within the PDX1 homeodomain.

## 3. Results

All findings presented follow the standardized interpretation framework defined in Section 2, with destabilizing variants consistently identified based on negative ΔΔG values and concordant structural perturbations.

### 3.1. Retrieval and Filtering of PDX1 nsSNPs

A comprehensive search of the dbSNP database identified 4434 single-nucleotide polymorphisms (SNPs) within the coding region of the PDX1 gene. Following annotation and filtering, only 403 nsSNPs resulting in amino acid substitutions were retained for further analysis. Variants with incomplete protein-level information were excluded. Minor allele frequency data indicated that the majority of retained nsSNPs were rare variants, suggesting a higher likelihood of functional relevance.

### 3.2. Results of Functional Impact Prediction of nsSNPs

The functional consequences of PDX1 nsSNPs were evaluated using SIFT, PolyPhen-2, FATHMM, and PROVEAN. A subset of 91 variants was consistently predicted to be deleterious across these multiple tools, indicating a high probability of functional disruption (Supplementary Table S1). These variants were prioritized for subsequent analyses. Variants predicted as tolerated or benign by most tools were considered less likely to exert significant biological effects.

### 3.3. Disease Association Analysis Results

Disease relevance of the prioritized nsSNPs was assessed using SNPs & GO and PhD-SNP. Several variants (55 nsSNPs) were classified as disease-associated by both tools, accompanied by moderate to high Reliability Index (RI) scores, reflecting strong confidence in the predictions (Supplementary Table S2). Variants predicted as neutral with low RI values were deprioritized.

### 3.4. Effect of nsSNPs on Protein Stability

Protein stability analysis using I-Mutant and MuPro revealed that most high-risk (41 nsSNPs) were associated with decreased protein stability. These findings were further supported by DynaMut2 analysis, which demonstrated alterations in protein flexibility and vibrational entropy in 27 nsSNPs (Supplementary Table S3). Collectively, these results suggest that the selected mutations destabilize the PDX1 protein structure.

### 3.5. Structural and Functional Consequence Analysis Results

MutPred2 analysis indicated that the prioritized 18 nsSNPs may disrupt key molecular features, including secondary structure elements, functional motifs, and potential post-translational modification sites (Table 1). Structural interpretation using the HOPE server revealed that several substitutions (7 nsSNPs) involved changes in residue size, charge, or hydrophobicity, potentially affecting protein folding and local structural integrity, particularly within conserved regions (Table 2).

### 3.6. Three-Dimensional Structural Modeling

**Figure 2:**
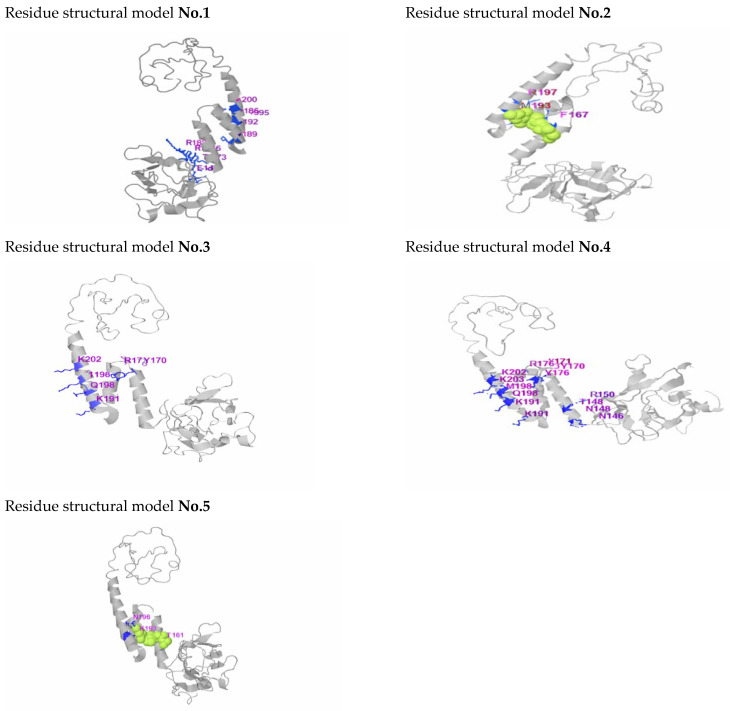
Structural model highlighting critical binding-site residues in the *PDX1* protein generated by I-TASSER (structural models: no. 1–5 as listed in Table 3).

**Figure 3:**
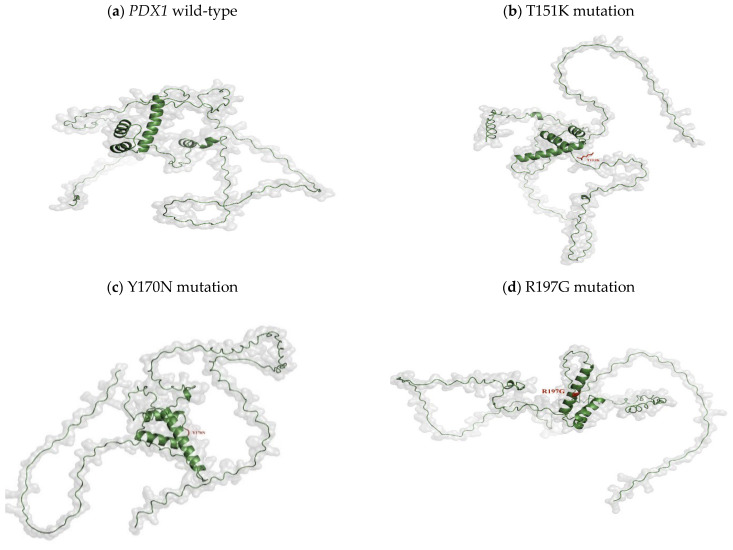
Structural impact of *PDX1* wild-type and missense variants on DNA binding. Three-dimensional structural models of wild-type and mutant PDX1 generated and visualized using PyMOL v2.0. Wild-type and mutant *PDX1*–DNA complexes (T151K, Y170N, R197G) shown as surface and cartoon representations. Mutated residues are highlighted as spheres. The T151K, Y170N, and R197G variants induce local conformational changes in the homeodomain, including altered side-chain orientation, residue packing, and electrostatic properties at the DNA-binding interface. R197G removes a DNA-contacting arginine, T151K adds a positive charge, and Y170N eliminates an aromatic stabilizing residue, indicating likely disruption of transcriptional activity.

The three-dimensional structure of the wild-type PDX1 protein was successfully modeled using I-TASSER. Mutant models were generated by introducing selected amino acid substitutions into the wild-type structure. The predicted ligand-binding residues for PDX1, as generated by I-TASSER, are listed for the top-ranked structural models (Table 3) and (Figure 2). Based on I-TASSER results and the seven nsSNPs identified through HOPE analysis (Table 2), the T151K, Y170N, and R197G mutations were selected for further investigation, as these missense variants are located within or in close proximity to the ligand-binding site residues. Comparative structural analysis using PyMOL (v.2) revealed noticeable conformational deviations in mutant proteins, including local structural rearrangements and altered residue orientations, especially within functionally important regions (Figure 3).

### 3.7. Molecular Dynamics Simulation Analysis

In order to be analyzed using MD simulations, variants were selected based on predefined quantitative thresholds. Using the combined criteria, T151K, Y170N, and R197G were ranked as the highest deleterious nsSNPs and therefore were prioritized for molecular dynamics analysis.

**Figure 4:**
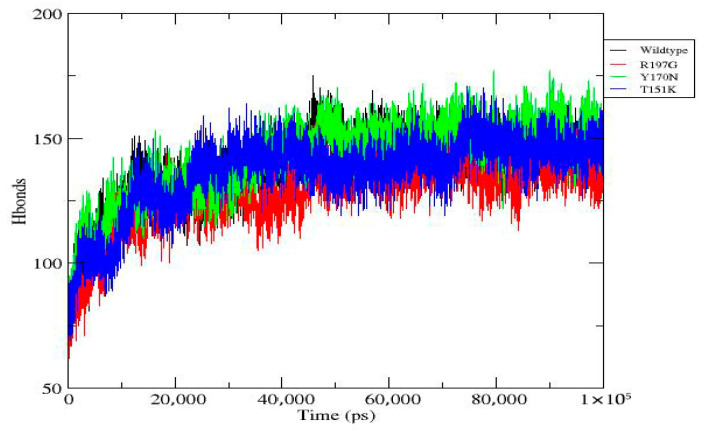
Hydrogen bond dynamics of wild-type and mutant *PDX1* proteins. Time evolution of hydrogen bonds over 100 ns of molecular dynamics simulations for the wild-type protein and R197G, Y170N, and T151K mutants. All systems exhibit rapid initial formation of hydrogen bonds followed by stabilization, indicating structural equilibration. R197G consistently shows fewer hydrogen bonds, suggesting reduced stability. Y170N exhibits the highest hydrogen-bond count, indicating enhanced intramolecular interactions, and T151K displays intermediate behavior similar to the wild type.

**Figure 5:**
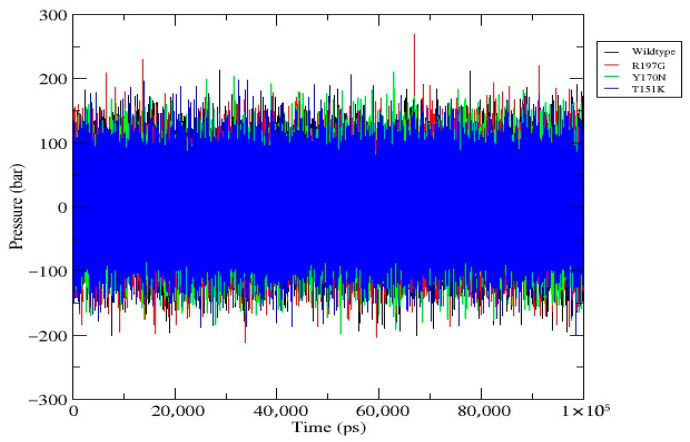
Pressure profile of wild-type and mutant *PDX1* proteins. Pressure fluctuations of all systems under NPT conditions during the 100 ns simulation. Pressures fluctuate around ~1 bar with no long-term drift, confirming effective pressure coupling and stable thermodynamic equilibration. Comparable profiles across all systems indicate that structural differences are mutation-specific and not due to pressure instability.

**Figure 6:**
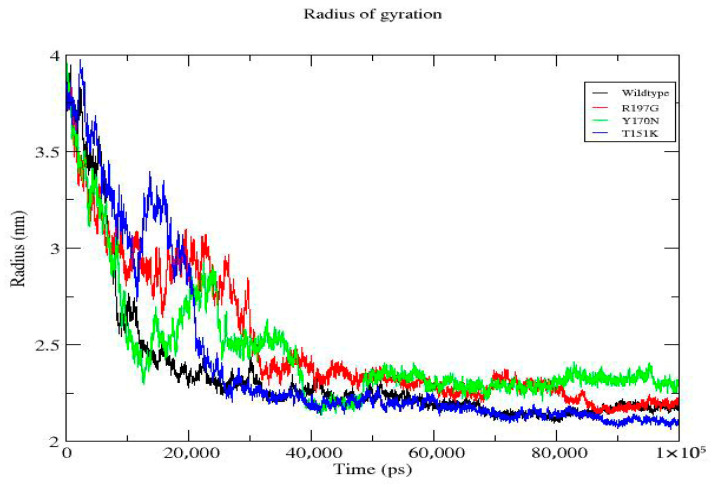
Radius of gyration (Rg) of wild-type and mutant *PDX1* proteins. Time-dependent radius of gyration during the 100 ns simulation. All proteins show rapid initial compaction followed by stabilization. R197G exhibits slightly higher Rg, indicating reduced compactness; T151K shows the lowest Rg, reflecting enhanced compactness; and Y170N displays intermediate behavior relative to the wild type.

**Figure 7:**
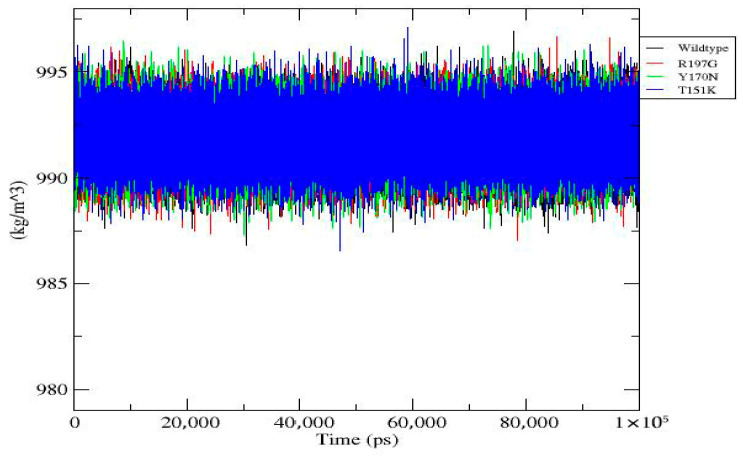
Density profile of wild-type and mutant *PDX1* proteins. Density values of wild-type and mutants of the *PDX1* protein remain stable throughout the 100 ns simulation, fluctuating narrowly around ~990–995 kg/m^3^. Strong overlap among profiles indicates that the introduced mutations do not perturb global density or volume equilibration.

**Figure 8:**
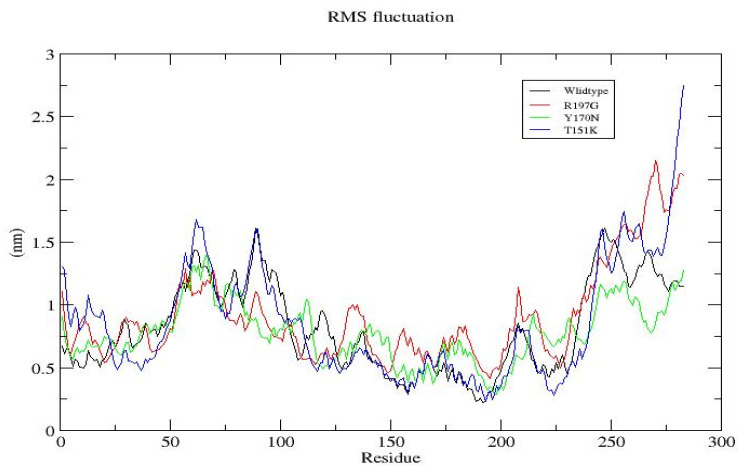
Residue-Level Flexibility (RMSF) of wild-type and mutant *PDX1* proteins. RMSF profiles show low flexibility in the protein core and higher mobility at terminal and loop regions. R197G and T151K exhibit increased local flexibility, while Y170N shows reduced fluctuations, suggesting enhanced local stability. These patterns are consistent with RMSD trends, where the wild type shows greater global conformational rearrangements, and mutants stabilize at lower RMSD values.

**Figure 9:**
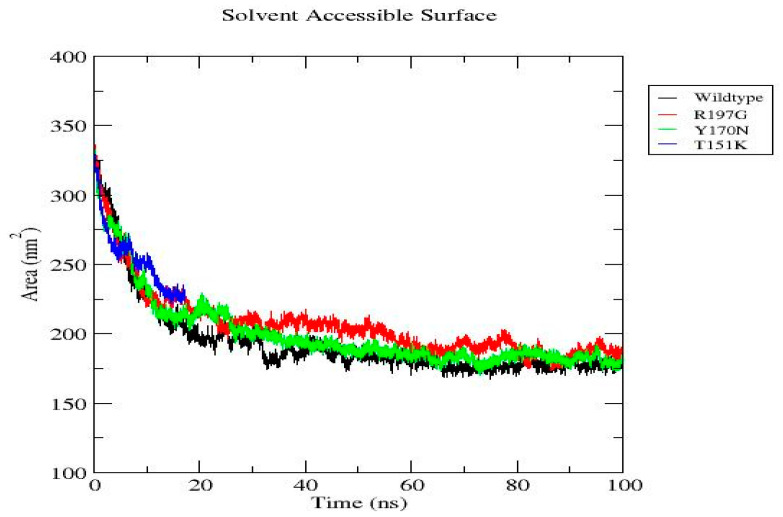
Solvent Accessible Surface Area (SASA) of wild-type and mutant *PDX1* proteins. SASA decreases rapidly during early simulation, reflecting protein compaction. After stabilization, the wild-type protein maintains the lowest SASA, whereas all mutants display higher solvent exposure, with R197G showing the greatest surface exposure, indicating altered conformational dynamics relative to the wild type.

**Figure 10:**
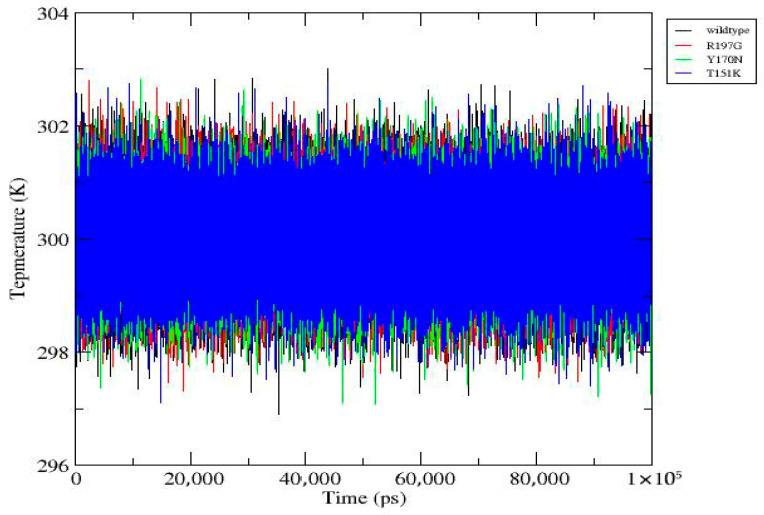
Temperature profile of wild-type and mutant *PDX1* proteins. Temperature remains tightly regulated around ~300 K for all systems throughout the 100 ns simulation, with only minor, random fluctuations. This confirms effective thermal control and ensures that observed structural differences are not influenced by thermal instability.

**Figure 11:**
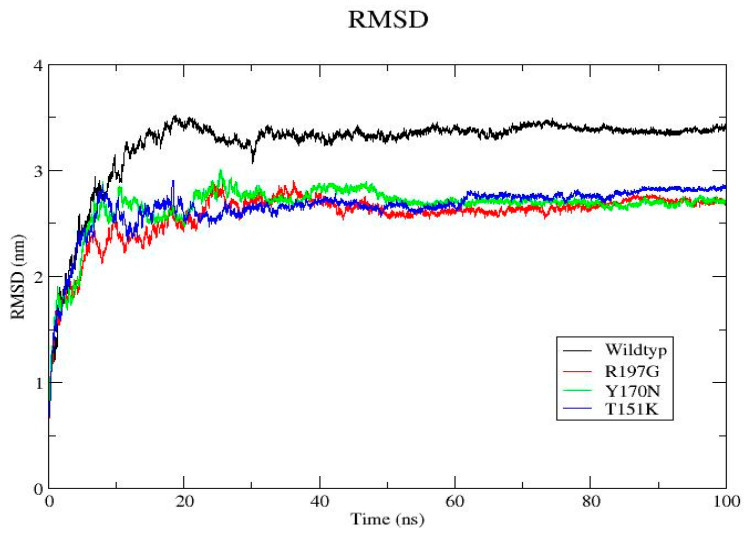
Backbone RMSD of wild-type and mutant *PDX1* proteins. Backbone RMSD over 100 ns shows rapid initial equilibration followed by stabilization after ~15–20 ns. The wild-type protein exhibits higher RMSD values, reflecting larger global conformational rearrangements, whereas R197G, Y170N, and T151K mutants stabilize at lower RMSD levels, indicating more constrained and stable conformations.

MD simulations were performed for the wild-type PDX1 protein and selected mutant variants over a 100 ns timeframe using GROMACS v2020.6. RMSD analysis showed that mutant proteins exhibited greater structural deviations compared to the wild type, indicating reduced stability. RMSF analysis demonstrated increased flexibility at mutation sites and neighboring residues. During the final 100 ns, the wild-type system exhibited an average RMSD of 0.23 ± 0.02 nm, whereas the R197G variant showed 0.31 ± 0.03 nm, indicating increased structural deviation. Additional parameters, including radius of gyration, solvent-accessible surface area, hydrogen bond formation, and secondary structure content, further supported the destabilizing effects of the mutations. Overall, the mutants displayed compromised structural compactness and altered dynamic behavior relative to the wild-type protein (Figure 4, Figure 5, Figure 6, Figure 7, Figure 8, Figure 9, Figure 10 and Figure 11) and (Table 4).

### 3.8. Secondary Structure Analysis Results

**Figure 12:**
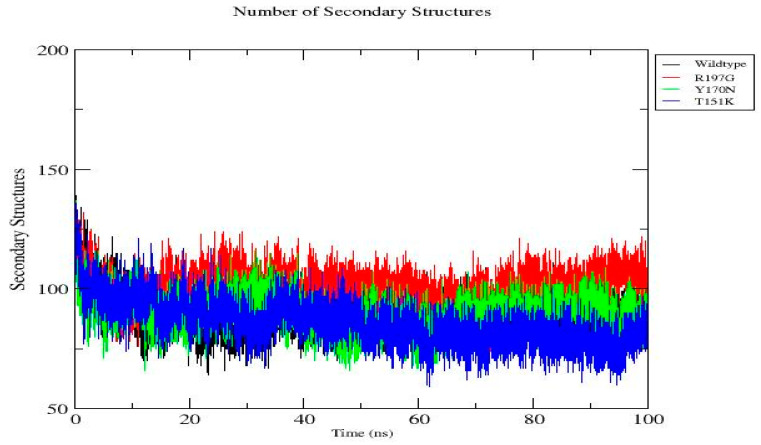
Time-dependent evolution of secondary structure content for the wild-type of *PDX1* protein and the R197G, Y170N, and T151K mutants during the 100 ns molecular dynamics simulation. Secondary structure elements were quantified using DSSP analysis and monitored throughout the simulation to assess mutation-induced changes in structural stability. The wild-type protein exhibits stable secondary structure content over time, whereas the mutants display distinct fluctuation patterns, reflecting mutation-specific effects on folding stability and conformational dynamics.

Quantitative DSSP analysis was performed to evaluate the impact of mutations on the *PDX1* protein secondary structure stability (Figure 12). Statistical averaging over the equilibrated portion of the trajectories (last 20 ns) revealed clear mutation-dependent differences in secondary structure content. The wild-type protein maintained a stable secondary structure with an average of (89.81 ± 5.43) structured residues, indicating preserved folding throughout the simulation. The R197G mutant exhibited a markedly higher mean secondary structure content (104.27 ± 5.59), suggesting enhanced stabilization or increased ordering relative to the wild type. In contrast, the Y170N mutant displayed a secondary structure profile comparable to the wild type (90.11 ± 6.35), albeit with increased fluctuations, indicating moderate destabilization. Notably, the T151K mutant showed the lowest average number of secondary structural elements (78.40 ± 6.11), accompanied by elevated variability, consistent with significant disruption of secondary structure integrity (Table 5). Overall, these results demonstrate that the investigated mutations exert distinct effects on protein structural stability, with T151K inducing the most pronounced destabilization, while R197G appears to enhance secondary structure formation, potentially altering the protein’s conformational landscape.

## 4. Discussion

PDX1 is a master transcription factor that plays a fundamental role in pancreatic development and the maintenance of mature β-cell function. Given its central regulatory role, even subtle alterations in PDX1 structure or activity can have profound effects on insulin production and glucose homeostasis. In this study, an integrated in silico framework was applied to systematically evaluate the functional and structural consequences of nsSNPs in the PDX1 gene, with the aim of identifying variants with a high likelihood of pathogenicity.

Initial sequence-based analyses revealed that a subset of nsSNPs was consistently predicted to be deleterious by multiple functional prediction tools. Concordant predictions across SIFT, PolyPhen-2, FATHMM, and PROVEAN suggest that these amino acid substitutions are evolutionarily unfavorable and likely disrupt essential functional features of the PDX1 protein. Such consistency across independent algorithms strengthens confidence in the prioritization of these variants, as reliance on a single predictive method may lead to biased or uncertain conclusions.

Disease association analysis using SNPs & GO and PhD-SNP further supported the pathogenic relevance of the prioritized variants. Variants classified as disease-associated with moderate to high Reliability Index (RI) values indicate a strong likelihood of involvement in diabetes-related phenotypes. These findings are consistent with previous reports demonstrating that pathogenic PDX1 mutations are frequently associated with monogenic forms of diabetes, particularly MODY4, through impaired transcriptional regulation of β-cell–specific genes [5,8,9,10].

Protein stability analysis provides additional insight into the molecular mechanisms underlying variant pathogenicity. Hence, most high-risk nsSNPs were predicted to decrease protein stability, as indicated by I-Mutant and MuPro analyses. Destabilization of PDX1 may result in improper folding, reduced protein half-life, or impaired interaction with DNA and transcriptional cofactors. DynaMut2 analysis further revealed alterations in protein flexibility and vibrational entropy, suggesting that these substitutions may perturb the dynamic equilibrium required for normal PDX1 function.

Structural findings were interpreted in direct alignment with standardized stability metrics to maintain internal consistency. Variants exhibiting negative ΔΔG values and concordant conformational perturbations were consistently described as destabilizing. Functional implications were discussed cautiously, particularly with respect to potential effects on DNA-binding affinity and transcriptional regulation, while avoiding extrapolation beyond computational evidence.

Structural interpretation using MutPred2 and HOPE indicated that several prioritized nsSNPs are likely to affect conserved residues, secondary structural elements, or functional motifs. Changes in amino acid size, charge, or hydrophobicity were predicted to disrupt local structural environments, potentially interfering with DNA-binding capacity or transcriptional activity. Given that PDX1 contains a highly conserved homeodomain essential for target gene recognition, structural perturbations within or near this region may have particularly severe functional consequences.

Three-dimensional structural modeling and comparative analysis of wild-type and mutant proteins R197G, Y170N, and T151K revealed notable conformational deviations in mutant models. These structural changes were further substantiated by molecular dynamics simulations, which demonstrated increased structural instability, altered flexibility, and reduced compactness in mutant proteins compared with the wild-type. Parameters such as RMSD, RMSF, radius of gyration, solvent-accessible surface area, and hydrogen bond dynamics collectively indicated compromised structural integrity and altered dynamic behavior in the mutant systems. Such dynamic instability is likely to impair the ability of PDX1 to maintain stable interactions with DNA and regulatory partners.

Although molecular dynamics simulations suggested relative structural stabilization of the R197G variant, thermodynamic stability does not necessarily equate to functional competence in DNA-binding transcription factors. Subtle alterations in residue orientation, electrostatic interactions, or DNA-contact geometry may impair transcriptional regulation despite apparent structural compactness. This interpretation is aligned with established principles in structural biology, which emphasize that protein stability and functional activity are related but distinct properties [45,46].

In this study, all molecular dynamics simulations were performed on the apo (DNA-unbound) form of PDX1 to assess intrinsic mutation-induced structural stability independent of DNA-mediated stabilization. Analyzing the apo state allows evaluation of whether mutations intrinsically destabilize the homeodomain prior to DNA engagement, which is a prerequisite for functional DNA binding. Simulating the apo form supports the physiological interpretation because DNA binding requires a pre-organized and intrinsically stable homeodomain. Any mutation that disrupts this intrinsic stability is likely to impair its biological DNA-binding function.

Taken together, missense mutations in PDX1 are linked to a wide spectrum of diabetes phenotypes, ranging from pancreatic agenesis and neonatal diabetes to MODY4 and type 2 diabetes. This broad clinical heterogeneity underscores the pronounced sensitivity of PDX1 to amino acid substitutions, particularly within functionally critical domains of the protein. Accumulating evidence indicates that even subtle alterations in the PDX1 homeodomain can result in significant functional impairment [7,8,9]. Notably, several reported variants within this region, including T151M, E164D, and E178G/K, markedly reduce transcriptional activation despite preserved nuclear localization, demonstrating that minor structural perturbations at the protein–DNA interface can profoundly disrupt downstream gene regulation [47,48].

To contextualize our computational findings within existing clinical evidence, we compared the prioritized PDX1 variants with classifications reported in ClinVar and examined consistency with published functional studies. Variants previously described as pathogenic typically affect domains critical for DNA binding or transcriptional activation. Our molecular dynamics analyses identified structural and dynamic perturbations that are mechanistically consistent with impaired transcription factor function, although we do not equate these changes directly with confirmed pathogenicity. Experimental studies done by Wang et al. (2019), published in Molecular Metabolism [47], and another study conducted by Yang et al. (2025), published in JCI Insight [48], demonstrate that missense variants in PDX1 can impair β-cell development, chromatin association, and protein–protein interactions. Collectively, our study provides structural-dynamic evidence that complements established variant interpretation frameworks, such as those proposed by the American College of Medical Genetics and Genomics [49], while emphasizing that functional validation remains essential for definitive clinical classification.

From a clinical standpoint, the identification and characterization of structurally and functionally disruptive PDX1 variants have important implications for the diagnosis and management of monogenic diabetes. Accurate interpretation of pathogenic variants can aid in distinguishing MODY4 from other forms of diabetes, thereby informing personalized treatment strategies and enabling appropriate genetic counseling. Furthermore, computational prioritization of high-risk missense variants represents a low-cost, effective approach for narrowing candidate mutations, guiding experimental validation, and focusing clinical attention on only variants that are most likely to exert significant biological and clinical effects.

Inclusively, this study demonstrates the value of integrating sequence-based pathogenicity prediction, structural modeling, and molecular dynamics simulations to systematically evaluate the functional impact of PDX1 nsSNPs. By combining these complementary in silico approaches, our study provides mechanistic insights into how specific missense variants may destabilize PDX1 structure, alter its dynamic behavior, and impair transcriptional function. Such structural and functional perturbations offer a plausible molecular basis for β-cell dysfunction and the development of diabetes. Collectively, these findings refine current understanding of PDX1 variants’ pathogenicity and establish a rational framework for prioritizing candidate mutations for experimental validation, while underscoring the expanding role of computational analyses in genetic variant interpretation and precision medicine.

## 5. Conclusions

The present study employed a comprehensive computational strategy to investigate the structural and functional consequences of non-synonymous SNPs in the *PDX1* gene. By integrating multiple bioinformatics tools with MD simulations, several high-risk variants were identified that are likely to impair protein stability, structural integrity, and dynamic behavior. These alterations may compromise the transcriptional regulatory functions of *PDX1* and contribute to β-cell dysfunction underlying MODY4 and related diabetic phenotypes. These findings underscore the value of integrated in silico analyses for prioritizing pathogenic variants and enhancing understanding of genotype–phenotype relationships. Future experimental and clinical studies are required to validate the predicted effects of the identified variants and to explore their potential implications for genetic diagnosis and personalized management of monogenic diabetes.

## 6. Study Limitations

While integrated computational approaches enhance predictive robustness, the functional consequences of identified PDX1 variants remain theoretical and require experimental validation through biochemical and cellular assays.

Although this study provides comprehensive insights into the structural and functional consequences of PDX1 missense variants, several limitations should be acknowledged. First, the analyses were based exclusively on computational predictions and MD simulations, which, despite their robustness, cannot fully capture the complexity of intracellular environments, post-translational regulation, or tissue-specific effects. Second, the structural models were generated using homology-based approaches, which may not fully reflect native conformations, particularly in flexible or intrinsically disordered regions of the protein. Third, population-level clinical data correlating specific variants with phenotypic severity were not incorporated, limiting direct genotype–phenotype inference. Consequently, the predicted pathogenic effects should be interpreted as hypotheses that require experimental validation using cellular, biochemical, or patient-derived models.

To address this limitation, we propose the following future analyses: protein–DNA docking of wild-type and mutant PDX1 homeodomains with insulin promoter DNA using established docking platforms (e.g., HADDOCK or similar protein–DNA docking tools). Comparative binding energy estimation to quantify mutation-induced changes in DNA-binding affinity. MD simulations of the protein–DNA complex to evaluate interface stability, hydrogen bond persistence, and recognition helix positioning.

## 7. Future Perspectives

Future studies should focus on experimental validation of the prioritized PDX1 variants using in vitro and in vivo systems to confirm their effects on protein stability, DNA-binding affinity, transcriptional activity, and β-cell function. Integration of functional assays with patient clinical data and population-scale genomic datasets will further enhance variant classification and clinical relevance. Additionally, extending MD simulations to longer timescales and incorporating protein–DNA or protein–cofactor complexes may provide deeper insight into regulatory mechanisms disrupted by pathogenic variants. From a translational perspective, systematic characterization of PDX1 mutations could improve genetic diagnosis of MODY4 and support precision medicine approaches by informing prognosis and therapeutic decision-making.

## Figures and Tables

**Table 1:** Pathogenicity prediction of PDX1 gene nsSNPs using MutPred2.

SNP Information			MutPred2	
SNP ID	Alleles	a. a mutation *	SCORE	Function affected
rs80356662	G>A	E178K	0.864	Loss of Allosteric site at R176
rs773768784	C>A	R198S	0.871	Loss of Helix, Loss of Allosteric site at R198, Loss of SUMOylation at K203
rs773768784	C>T	R198C	0.823	Loss of Helix, Loss of Allosteric site at R198, Loss of SUMOylation at K203
rs1555241857	A>C	N168H	0.738	Loss of Acetylation at K169
rs1957810066	G>C	R198P	0.944	Loss of Helix, Loss of Allosteric site at R198, Loss of SUMOylation at K203
rs1957810066	G>T	R198L	0.861	Loss of Allosteric site at R198. Loss of SUMOylation at K203
rs2500206329	G>A	R176Q	0.801	Loss of Allosteric site at R176
rs202159230	T>G	L185W	0.878	Loss of Helix
rs565726855	C>A	T151K	0.890	Loss of Methylation at K147, Loss of Proteolytic cleavage at R150, Loss of Ubiquitylation at K147
rs765466519	T>A	Y170N	0.923	Loss of Acetylation at K169. Loss of Allosteric site at R175
rs948183052	G>T	L185F	0.784	Loss of Helix
rs1229246846	C>G	R197G	0.878	Loss of Allosteric site at R197, Loss of Helix, Loss of SUMOylation at K202
rs1245215769	G>A	R188K	0.814	Loss of Allosteric site at R188. Loss of N-linked glycosylation at N184
rs1278981936	C>G	C18W	0.750	Loss of Intrinsic disorder, Loss of Phosphorylation at Y14, Loss of Sulfation at Y14
rs2137506152	C>T	R173W	0.859	Loss of Phosphorylation at Y14, Loss of Sulfation at Y14
rs2500206228	T>A	F165Y	0.481	Loss of Acetylation at K169. Loss of Allosteric site at R176
rs2500206438	G>A	E187K	0.896	Loss of Strand, Loss of Allosteric site at R197
rs2500206466	G>C	W193C	0.938	Loss of Phosphorylation at Y8. Loss of Sulfation at Y7

* a. a mutation: amino acid mutation.

**Table 2:** The PDX1 nsSNPs identified as the most deleterious based on the HOPE tool.

SNP Information			HOPE			
SNP ID	alleles	a. a mutation *	Size	Hydrophobicity	Key structural impact	The variant’s MetaRNN Score
rs1555241857	A>C	N168H	Increased	--	The mutant residue is bigger; this might lead to bumps.	0.9332391
rs202159230	T>G	L185W	Increased	--	The mutant residue is bigger; this might lead to bumps.	0.945027
rs565726855	C>A	T151K	Increased	Differs	The mutant residue is bigger; this might lead to bumps.	0.92307395
rs765466519	T>A	Y170N	Decreased	Differs	The mutant residue is smaller; this might lead to loss of interaction. Hydrophobic interaction, either in the core of the protein or on the surface, will be lost.	0.9613937
rs948183052	G>T	L185F	Increased	--	The mutant residue is bigger; this might lead to bumps.	0.945027
rs1229246846	C>G	R197G	Decreased	Differs	The mutant residue is smaller; this might lead to loss of interactions. The mutation introduces a more hydrophobic residue at this position. This can result in loss of hydrogen bonds and/or disturb correct folding.	0.97457975
rs1278981936	C>G	C18W	Increased	--	The mutant residue is bigger; this might lead to bumps.	0.9024972

* a. a mutation: amino acid mutation.

**Table 3:** Structural models of the PDX1 protein generated by I-TASSER.

Rank	C-score	Cluster Size	PDB Hit	Ligand Name	Download Complex	Predicted Ligand-Binding Site Residues
1	0.2	24	1zq3P	Nuc. Acid	N/A	145, 148, 149, 150, 151, 153, 189, 192, 195, 196, 200
2	0.1	12	1tkcB	M6T	Rep, Mult	167, 193, 197
3	0.08	9	2hddB	Nuc. Acid	N/A	170, 176, 191, 195, 198, 202
4	0.06	7	1ahdP	Nuc. Acid	N/A	146, 147, 148, 149, 150, 170, 171, 173, 176, 191, 195, 198, 199, 202, 203
5	0.04	5	1itzB	TPP	N/A	161, 193, 196

**Table 4:** Molecular dynamics simulation parameters and comparative outcomes. Key dynamic and structural metrics derived from 100 ns molecular dynamics simulations of wild-type and mutant PDX1 proteins.

Figure	Title/Reference	Key Findings	Units/Measures	Suggested Color Scheme
Figure 4	Hydrogen Bond Dynamics	All systems reach stable hydrogen-bond networks. R197G shows reduced hydrogen bonding, Y170N shows enhanced interactions, and T151K is similar to wild type.	Number of H-bonds	Wild-type: blue; R197G: red; Y170N: green; T151K: orange
Figure 5	Pressure Profile	Pressure fluctuates around ~1 bar for all systems with no drift, confirming thermodynamic stability.	Pressure (bar)	Wild-type: blue; R197G: red; Y170N: green; T151K: orange
Figure 6	Radius of Gyration (Rg)	All systems compact early and stabilize. R197G shows slightly higher Rg (less compact), T151K is the most compact, and Y170N is intermediate.	Radius of gyration (nm)	Wild-type: blue; R197G: red; Y170N: green; T151K: orange
Figure 7	Density Profile	Stable density (~990–995 kg/m^3^) for all systems; mutations do not affect global density.	Density (kg/m^3^)	Wild-type: blue; R197G: red; Y170N: green; T151K: orange
Figure 8	Root Mean Square Fluctuation (RMSF)	Core residues are stable; terminal/loop regions show higher flexibility. R197G and T151K have increased local flexibility; Y170N shows reduced fluctuations.	RMSF (nm)	Wild-type: blue; R197G: red; Y170N: green; T151K: orange
Figure 9	Solvent Accessible Surface Area (SASA)	Early decrease reflects compaction. Wild type has the lowest SASA; mutants have higher; R197G is the most solvent-exposed.	SASA (nm^2^)	Wild-type: blue; R197G: red; Y170N: green; T151K: orange
Figure 10	Temperature Profile	Temperature stable around ~300 K with minor fluctuations; thermal regulation is effective.	Temperature (K)	Wild-type: blue; R197G: red; Y170N: green; T151K: orange
Figure 11	Backbone RMSD	All systems stabilize after ~15–20 ns. Wild type shows larger global rearrangements; mutants stabilize at lower RMSD, indicating constrained conformations.	RMSD (nm)	Wild-type: blue; R197G: red; Y170N: green; T151K: orange

**Table 5:** Average number of secondary structural elements obtained from DSSP analysis over the final 20 ns of MD simulations of *PDX1* protein.

System	Secondary Structures (Mean ± SD)
Wild type	89.81 ± 5.43
R197G	104.27 ± 5.59
Y170N	90.11 ± 6.35
T151K	78.40 ± 6.11

## Data Availability

The original contributions presented in the study are included in the article/Supplementary Materials. Further inquiries can be directed to the corresponding author.

## Supplementary Materials

The following supporting information can be downloaded at: https://www.mdpi.com/article/10.3390/genes17030273/s1, Table S1: Prediction of nsSNPs’ effect on PDX1 protein structure and function using SIFT, PolyPhen2, FATHMM, and PROVEAN tools; Table S2: Prediction of PDX1 gene nsSNPs disease association using SNPS & GO and PhD-SNP tools; Table S3: Prediction of nsSNPs effect on PDX1 protein stability using I-Mutant2.0, MuPro, and DynaMut2 tools.
