# Supplementary material for: Integrated Computational Analysis Reveals Structurally Destabilizing Missense Variants in the PDX1 Transcription Factor
Source: Genes (Basel). 2026 Feb 27;17(3):273. doi: 10.3390/genes17030273 (PMC13026271; doi:10.3390/genes17030273)
Supplement: Supplementary file 1 [file genes-17-00273-s001.zip › genes-4163047-supplementary.pdf]

Supplementary Tables S1–S3

Supplementary Table S1. Prediction of nsSNPs' effect on PDX1 protein structure and function using SIFT, PolyPhen2, FATHMM, and PROVEAN tools.

SNP information				SIFT		POLYPHEN2		FATHMM		PROVEAN	
SNP ID	alleles	**a. a mutation ***	MAF**	**PREDICTION**	**SCORE**	**PREDICTION**	**SCORE**	**PREDICTION**	SCORE	**PREDICTION**	**SCORE**
rs80356661	G>T	E164D	T=0./0	AFFECT	0.00	probably damaging	1.000	DAMAGE	- 4.05	Deleterious	-2.817
rs80356662	G>A	E178K	A=0./0	AFFECT	0.00	probably damaging	1.000	DAMAGE	-4.04	Deleterious	-3.933
rs137852783	G>T	D76Y	A=0.00331/183	AFFECT	0.00	probably damaging	0.986	DAMAGE	-2.21	Deleterious	-3.049
rs137852785	T>C	C18R	C=0.000138/5	AFFECT	0.00	probably damaging	0.999	DAMAGE	-2.09	Deleterious	-4.443
rs137852785	T>G	C18G	C=0.000138/5	AFFECT	0.01	probably damaging	0.998	DAMAGE	-2.17	Deleterious	-3.911
rs137852786	G>A	R197H	A=0./0	AFFECT	0.00	probably damaging	1.000	DAMAGE	-4.43	Deleterious	-4.944
rs137852786	G>T	R197L	A=0./0	AFFECT	0.00	probably damaging	0.984	DAMAGE	-4.54	Deleterious	-6.922
rs192902098	C>A	P33T	G=0.00006/2	AFFECT	0.00	probably damaging	1.000	DAMAGE	-3.12	Deleterious	-4.321
rs192902098	C>T	P33S	G=0.00006/2	AFFECT	0.01	probably damaging	1.000	DAMAGE	-3.12	Deleterious	-4.129
rs193922355	C>G	R148G	A=0.000007/1	AFFECT	0.00	probably damaging	1.000	DAMAGE	-4.24	Deleterious	-6.806
rs193922356	A>C	K191Q	C=0./0	AFFECT	0.00	probably damaging	1.000	DAMAGE	-3.97	Deleterious	-3.956
rs387906777	A>G	E178G	NA***	AFFECT	0.00	probably damaging	1.000	DAMAGE	-4.17	Deleterious	-6.883
rs564129447	C>A	P71T	A=0.000304/7	AFFECT	0.03	probably damaging	0.985	DAMAGE	-2.72	Deleterious	-2.768
rs767363575	C>A	P33H	T=0.000028/1	AFFECT	0.00	probably damaging	1.000	DAMAGE	-3.14	Deleterious	-4.722
rs767363575	C>T	P33L	T=0.000028/1	AFFECT	0.00	probably damaging	1.000	DAMAGE	-3.11	Deleterious	-5.559
rs771543377	A>C	N196T	C=0.000002/1	AFFECT	0.00	probably damaging	1.000	DAMAGE	-5.81	Deleterious	-5.933
rs773768784	C>A	R198S	A=0.000008/1	AFFECT	0.00	probably damaging	1.000	DAMAGE	-5.50	Deleterious	-5.926
rs773768784	C>T	R198C	A=0.000008/1	AFFECT	0.00	probably damaging	1.000	DAMAGE	-5.51	Deleterious	-7.902
rs936861677	T>A	L36Q	G=0.00004/1	AFFECT	0.00	probably damaging	1.000	DAMAGE	-2.64	Deleterious	-2.714
rs936861677	T>C	L36P	G=0.00004/1	AFFECT	0.00	probably damaging	1.000	DAMAGE	-2.64	Deleterious	-2.941
rs1169068861	A>C	E162A	C=0./0	AFFECT	0.00	probably damaging	1.000	DAMAGE	-4.53	Deleterious	-5.600

rs1175289727	A>C	D226A	C=0.000002/1	AFFECT	0.00	probably damaging	1.000	DAMAGE	-2.73	Deleterious	-3.000
rs1333313782	A>T	D16V	T=0.0000007/1	AFFECT	0.02	probably damaging	0.982	DAMAGE	-2.31	Deleterious	-2.858
rs1342320098	A>G	D64G	NA	AFFECT	0.02	probably damaging	1.000	DAMAGE	-2.53	Deleterious	-3.388
rs1358532247	A>T	Q21L	T=0./0	AFFECT	0.00	probably damaging	1.000	DAMAGE	-2.30	Deleterious	-2.948
rs1555241857	A>C	N168H	NA	AFFECT	0.03	probably damaging	1.000	DAMAGE	-4.03	Deleterious	-4.383
rs1957809431	G>T	E178D	T=0./0	AFFECT	0.00	probably damaging	1.000	DAMAGE	-4.10	Deleterious	-2.950
rs1957810066	G>C	R198P	T=0./0	AFFECT	0.00	probably damaging	1.000	DAMAGE	-5.51	Deleterious	-6.914
rs1957810066	G>T	R198L	T=0./0	AFFECT	0.00	probably damaging	1.000	DAMAGE	-5.50	Deleterious	-6.914
rs2500200525	G>T	W121C	A=0.000001/1	AFFECT	0.00	probably damaging	1.000	DAMAGE	-3.28	Deleterious	-10.783
rs2500206125	C>T	R150C	NA	AFFECT	0.00	probably damaging	1.000	DAMAGE	-5.53	Deleterious	-7.778
rs2500206217	A>G	K163R	NA	AFFECT	0.03	probably damaging	1.000	DAMAGE	-3.96	Deleterious	-2.817
rs2500206329	G>A	R176Q	NA	AFFECT	0.00	probably damaging	1.000	DAMAGE	-4.39	Deleterious	-3.933
rs2500206458	T>C	I190T	NA	AFFECT	0.00	probably damaging	1.000	DAMAGE	-2.73	Deleterious	-4.944
rs202159230	T>G	L185W	G=0.000007/1	AFFECT	0.00	probably damaging	1.000	DAMAGE	-4.96	Deleterious	-5.933
rs565726855	C>A	T151K	T=0.000007/1	AFFECT	0.01	probably damaging	1.000	DAMAGE	-4.30	Deleterious	-5.833
rs565726855	C>T	T151M	T=0.000007/1	AFFECT	0.00	probably damaging	1.000	DAMAGE	-4.35	Deleterious	-5.833
rs747825143	C>G	P223A	T=0./0	AFFECT	0.05	probably damaging	0.978	DAMAGE	-2.96	Deleterious	-2.607
rs761107416	A>T	D206V	T=0.000008/1	AFFECT	0.01	probably damaging	0.983	DAMAGE	-2.69	Deleterious	-3.206
rs764675125	C>A	H128Q	G=0./0	AFFECT	0.02	probably damaging	1.000	DAMAGE	-2.74	Deleterious	-5.555
rs764810731	A>G	M122V	T=0.00001/1	AFFECT	0.00	probably damaging	0.997	DAMAGE	-2.97	Deleterious	-3.363
rs765466519	T>A	Y170N	A=0./0	AFFECT	0.00	probably damaging	1.000	DAMAGE	-4.27	Deleterious	-8.539
rs771445149	G>A	R209H	A=0./0	AFFECT	0.01	probably damaging	1.000	DAMAGE	-2.77	Deleterious	-3.683
rs2500199386	G>A	C35Y	A=0.0000007/1	AFFECT	0.00	probably damaging	1.000	DAMAGE	-2.41	Deleterious	-5.725
rs2500199821	A>G	Y68C	G=0.000001/1	AFFECT	0.00	probably damaging	0.970	DAMAGE	-2.66	Deleterious	-3.340
rs2500200512	C>A	F119L	NA	AFFECT	0.00	probably damaging	0.996	DAMAGE	-2.89	Deleterious	-4.845

rs2500200523	T>A	W121R	A=0.000002/1	AFFECT	0.00	probably damaging	1.000	DAMAGE	-3.25	Deleterious	-11.653
rs2500200544	G>T	M122I	T=0.000002/1	AFFECT	0.01	probably damaging	0.997	DAMAGE	-3.01	Deleterious	-3.337
rs2500200548	T>C	S124P	C=0.000003/2	AFFECT	0.02	probably damaging	0.998	DAMAGE	-2.89	Deleterious	-2.948
rs2500200554	C>T	T125I	T=0.000004/3	AFFECT	0.03	probably damaging	1.000	DAMAGE	-2.66	Deleterious	-4.376
rs2500206074	A>C	K147Q	NA	AFFECT	0.00	probably damaging	1.000	DAMAGE	-4.20	Deleterious	-3.889
rs2500206074	A>G	K147E	NA	AFFECT	0.00	probably damaging	1.000	DAMAGE	-4.17	Deleterious	-3.889
rs2500206127	G>T	R150L	T=0.000002/1	AFFECT	0.01	probably damaging	1.000	DAMAGE	-5.50	Deleterious	-6.806
rs2500206160	C>A	T154K	A=0.0000007/1	AFFECT	0.00	probably damaging	1.000	DAMAGE	-4.13	Deleterious	-5.333
rs2500206199	T>G	L159R	G=0.0000007/1	AFFECT	0.03	probably damaging	1.000	DAMAGE	-3.85	Deleterious	-5.633
rs2500206228	T>A	F165Y	A=0.000001/1	AFFECT	0.01	probably damaging	1.000	DAMAGE	-5.56	Deleterious	-2.817
rs2500206314	G>T	R173L	T=0.000001/1	AFFECT	0.01	probably damaging	1.000	DAMAGE	-3.93	Deleterious	-6.875
rs2500206326	C>T	R176W	T=0.000001/1	AFFECT	0.01	probably damaging	1.000	DAMAGE	-4.41	Deleterious	-7.867
rs2500206364	C>G	A180G	G=0.000001/1	AFFECT	0.00	probably damaging	1.000	DAMAGE	-4.86	Deleterious	-3.884
rs2500206438	G>A	E187K	A=0.000001/1	AFFECT	0.00	probably damaging	1.000	DAMAGE	-4.13	Deleterious	-3.862
rs2500206466	G>C	W193C	NA	AFFECT	0.00	probably damaging	1.000	DAMAGE	-6.94	Deleterious	-12.856
rs2500206510	G>T	W201L	T=0.000002/1	AFFECT	0.05	probably damaging	0.993	DAMAGE	-3.96	Deleterious	-11.091
rs2500206527	A>G	E204G	G=0.000002/1	AFFECT	0.00	probably damaging	0.995	DAMAGE	-3.80	Deleterious	-5.335
rs771445149	G>C	R209P	A=0./0	AFFECT	0.00	probably damaging	0.998	DAMAGE	-2.79	Deleterious	-3.978
rs771445149	G>T	R209L	A=0./0	AFFECT	0.02	probably damaging	0.983	DAMAGE	-2.73	Deleterious	-4.622
rs781025559	G>T	G137C	T=0./0	AFFECT	0.04	probably damaging	1.000	DAMAGE	-3.84	Deleterious	-3.649
rs948183052	G>T	L185F	A=0./0	AFFECT	0.00	probably damaging	1.000	DAMAGE	-4.94	Deleterious	-3.956
rs1045275852	C>G	R209G	T=0./0	AFFECT	0.01	probably damaging	0.983	DAMAGE	-2.73	Deleterious	-4.606
rs1045275852	C>T	R209C	T=0./0	AFFECT	0.00	probably damaging	1.000	DAMAGE	-2.82	Deleterious	-4.941
rs1055958773	G>C	K203N	C=0./0	AFFECT	0.00	probably damaging	1.000	DAMAGE	-4.13	Deleterious	-4.899
rs1210186252	C>G	R22G	NA	AFFECT	0.00	probably damaging	0.999	DAMAGE	-2.31	Deleterious	-2.684

rs1229246846	C>G	R197G	G=0./0	AFFECT	0.00	probably damaging	0.992	DAMAGE	-4.54	Deleterious	-6.922
rs1245215769	G>A	R188K	C=0./0	AFFECT	0.01	probably damaging	0.998	DAMAGE	-3.95	Deleterious	-2.792
rs1245215769	G>C	R188T	C=0./0	AFFECT	0.04	probably damaging	1.000	DAMAGE	-4.03	Deleterious	-5.600
rs1245281170	C>T	P32L	T=0.000007/1	AFFECT	0.01	probably damaging	1.000	DAMAGE	-2.55	Deleterious	-4.782
rs1278981936	C>G	C18W	A=0./0	AFFECT	0.00	probably damaging	1.000	DAMAGE	-2.21	Deleterious	-4.205
rs1297553641	C>T	S172L	T=0./0	AFFECT	0.00	probably damaging	1.000	DAMAGE	-4.04	Deleterious	-5.035
rs1300494277	C>T	P67L	T=0.0000015/2	AFFECT	0.01	probably damaging	1.000	DAMAGE	-2.54	Deleterious	-3.864
rs1352474312	C>A	P71H	G=0./0	AFFECT	0.02	probably damaging	0.999	DAMAGE	-2.76	Deleterious	-2.840
rs1352474312	C>G	P71R	G=0./0	AFFECT	0.02	probably damaging	0.995	DAMAGE	-2.75	Deleterious	-3.188
rs1384588250	C>T	S231F	NA***	AFFECT	0.00	probably damaging	0.999	DAMAGE	-2.81	Deleterious	-3.491
rs1456103730	G>T	R155L	T=0.0000029/4	AFFECT	0.00	probably damaging	1.000	DAMAGE	-2.76	Deleterious	-6.206
rs1466692234	G>T	D226Y	A=0.000031/1	AFFECT	0.00	probably damaging	1.000	DAMAGE	-2.28	Deleterious	-4.483
rs1478081393	T>G	W134G	G=0.000002/1	AFFECT	0.02	probably damaging	0.988	DAMAGE	-2.58	Deleterious	-8.402
rs1957771331	A>G	Y8C	G=0./0	AFFECT	0.01	probably damaging	1.000	DAMAGE	-2.18	Deleterious	-3.935
rs1957777698	T>A	M122K	A=0.000031/1	AFFECT	0.00	probably damaging	0.999	DAMAGE	-3.05	Deleterious	-5.170
rs1957809088	T>C	F167L	C=0./0	AFFECT	0.01	probably damaging	1.000	DAMAGE	-3.87	Deleterious	-5.633
rs1957809157	C>A	N168K	G=0.000031/1	AFFECT	0.01	probably damaging	0.999	DAMAGE	-4.01	Deleterious	-5.167
rs2137506152	C>T	R173W	NA	AFFECT	0.00	probably damaging	1.000	DAMAGE	-4.02	Deleterious	-7.858
rs2500198954	A>G	Y14C	G=0.000001/1	AFFECT	0.00	probably damaging	0.999	DAMAGE	-2.17	Deleterious	-3.443
rs2500199385	T>G	C35G	G=0.0000075/10	AFFECT	0.00	probably damaging	0.999	DAMAGE	-2.40	Deleterious	-6.053

*a. a mutation: amino acid mutation **MAF: Minor Allele Frequency ***NA: not available.

Supplementary Table S2. Prediction of PDX1 gene nsSNPs disease association using SNPS & GO and PhD-SNP tools.

SNP information				PhD-SNP		SNP and GO	
SNP ID	alleles	a. a mutation*	MAF**	EFFECT	RI***	EFFECT	RI***
rs80356661	G>T	E164D	T=0./0	DISEASE	6	DISEASE	9
rs80356662	G>A	E178K	A=0./0	DISEASE	5	DISEASE	9
rs137852785	T>C	C18R	C=0.000138/5	DISEASE	4	DISEASE	8
rs137852785	T>G	C18G	C=0.000138/5	DISEASE	4	DISEASE	6
rs137852786	G>A	R197H	A=0./0	DISEASE	7	DISEASE	8

rs137852786	G>T	R197L	A=0./0	DISEASE	6	DISEASE	9
rs193922355	C>G	R148G	A=0.000007/1	DISEASE	2	DISEASE	9
rs773768784	C>A	R198S	A=0.000008/1	DISEASE	9	DISEASE	7
rs773768784	C>T	R198C	A=0.000008/1	DISEASE	6	DISEASE	7
rs1169068861	A>C	E162A	C=0./0	DISEASE	3	DISEASE	8
rs1555241857	A>C	N168H	NA****	DISEASE	1	DISEASE	7
rs1957809431	G>T	E178D	T=0./0	DISEASE	5	DISEASE	8
rs1957810066	G>C	R198P	T=0./0	DISEASE	9	DISEASE	8
rs1957810066	G>T	R198L	T=0./0	DISEASE	6	DISEASE	8
rs2500200525	G>T	W121C	A=0.000001/1	DISEASE	8	DISEASE	9
rs2500206125	C>T	R150C	NA	DISEASE	7	DISEASE	9
rs2500206217	A>G	K163R	NA	DISEASE	4	DISEASE	7
rs2500206329	G>A	R176Q	NA	DISEASE	7	DISEASE	9
rs202159230	T>G	L185W	G=0.000007/1	DISEASE	6	DISEASE	9
rs565726855	C>A	T151K	T=0.000007/1	DISEASE	4	DISEASE	7
rs565726855	C>T	T151M	T=0.000007/1	DISEASE	2	DISEASE	4
rs764675125	C>A	H128Q	G=0./0	DISEASE	1	DISEASE	5
rs764675125	C>G	H128Q	G=0./0	DISEASE	1	DISEASE	5
rs764810731	A>G	M122V	T=0.00001/1	DISEASE	6	DISEASE	8
rs765466519	T>A	Y170N	A=0./0	DISEASE	1	DISEASE	9
rs771445149	G>C	R209P	A=0./0	DISEASE	3	DISEASE	7
rs781025559	G>T	G137C	T=0./0	DISEASE	8	DISEASE	2
rs948183052	G>T	L185F	A=0./0	DISEASE	5	DISEASE	9
rs1045275852	C>G	R209G	T=0./0	DISEASE	3	DISEASE	2
rs1045275852	C>T	R209C	T=0./0	DISEASE	3	DISEASE	5
rs1055958773	G>C	K203N	C=0. /0	DISEASE	4	DISEASE	7
rs1229246846	C>G	R197G	G=0. /0	DISEASE	8	DISEASE	8
rs1245215769	G>A	R188K	C=0. /0	DISEASE	6	DISEASE	9
rs1245215769	G>C	R188T	C=0. /0	DISEASE	1	DISEASE	9
rs1278981936	C>G	C18W	A=0. /0	DISEASE	6	DISEASE	7
rs1297553641	C>T	S172L	T=0. /0	DISEASE	4	DISEASE	7
rs1478081393	T>G	W134G	G=0.000002/1	DISEASE	3	DISEASE	5
rs1957771331	A>G	Y8C	G=0. /0	DISEASE	0	DISEASE	6
rs1957777698	T>A	M122K	A=0.000031/1	DISEASE	9	DISEASE	9
rs1957809088	T>C	F167L	C=0. /0	DISEASE	3	DISEASE	8
rs1957809157	C>A	N168K	G=0.000031/1	DISEASE	5	DISEASE	7
rs2137506152	C>T	R173W	NA	DISEASE	7	DISEASE	9
rs2500198954	A>G	Y14C	G=0.000001/1	DISEASE	1	DISEASE	7
rs2500200512	C>A	F119L	NA	DISEASE	6	DISEASE	4
rs2500200523	T>A	W121R	A=0.000002/1	DISEASE	9	DISEASE	9
rs2500200544	G>T	M122I	T=0.000002/1	DISEASE	5	DISEASE	8
rs2500200548	T>C	S124P	C=0.000003/2	DISEASE	7	DISEASE	7
rs2500206127	G>T	R150L	T=0.000002/1	DISEASE	3	DISEASE	9
rs2500206160	C>A	T154K	A=0.0000007/1	DISEASE	8	DISEASE	9
rs2500206199	T>G	L159R	G=0.0000007/1	DISEASE	5	DISEASE	8
rs2500206228	T>A	F165Y	A=0.000001/1	DISEASE	6	DISEASE	9
rs2500206314	G>T	R173L	T=0.000001/1	DISEASE	4	DISEASE	10
rs2500206326	C>T	R176W	T=0.000001/1	DISEASE	6	DISEASE	9
rs2500206438	G>A	E187K	A=0.000001/1	DISEASE	7	DISEASE	9

rs2500206466	G>C	W193C	NA	DISEASE	8	DISEASE	7

*a. a mutation: amino acid mutation **MAF: Minor Allele Frequency ***RI: Reliability Index ****NA: not available.

Supplementary Table S3. Prediction of nsSNPs effect on PDX1 protein stability using I-Mutant2.0, MuPro, and DynaMut2 tools.

SNP information			I-Mutant		MUPROT		DYNAMUT2	
SNP ID	alleles	a. a mutation*	stability	RI**	stability	RI**	Prediction	KCAL/MOL
rs80356661	G>T	E164D	DECREASE	3	DECREASE	3	DESTABILISING	-0.91
rs80356662	G>A	E178K	DECREASE	8	DECREASE	8	DESTABILISING	-0.44
rs193922355	C>G	R148G	DECREASE	4	DECREASE	4	DESTABILISING	-0.33
rs773768784	C>A	R198S	DECREASE	8	DECREASE	8	DESTABILISING	-2.28
rs773768784	C>T	R198C	DECREASE	6	DECREASE	6	DESTABILISING	-1.59
rs1555241857	A>C	N168H	DECREASE	9	DECREASE	9	DESTABILISING	-0.56
rs1957810066	G>C	R198P	DECREASE	8	DECREASE	8	DESTABILISING	-0.08
rs1957810066	G>T	R198L	DECREASE	7	DECREASE	7	DESTABILISING	-0.63
rs2500206217	A>G	K163R	DECREASE	4	DECREASE	4	DESTABILISING	-0.89
rs2500206329	G>A	R176Q	DECREASE	9	DECREASE	9	DESTABILISING	-0.98
rs202159230	T>G	L185W	DECREASE	8	DECREASE	8	DESTABILISING	-2.13
rs565726855	C>A	T151K	DECREASE	4	DECREASE	4	DESTABILISING	-0.26
rs764810731	A>G	M122V	DECREASE	6	DECREASE	6	DESTABILISING	-0.61
rs764810731	A>T	M122L	DECREASE	3	DECREASE	3	DESTABILISING	-1.42
rs765466519	T>A	Y170N	DECREASE	7	DECREASE	7	DESTABILISING	-0.39
rs948183052	G>T	L185F	DECREASE	9	DECREASE	9	DESTABILISING	-1.63
rs1045275852	C>G	R209G	DECREASE	6	DECREASE	6	DESTABILISING	-0.33
rs1229246846	C>G	R197G	DECREASE	8	DECREASE	8	DESTABILISING	-1.99
rs1245215769	G>A	R188K	DECREASE	9	DECREASE	9	DESTABILISING	-0.26
rs1278981936	C>G	C18W	DECREASE	6	DECREASE	6	DESTABILISING	-0.48
rs1478081393	T>G	W134G	DECREASE	8	DECREASE	8	DESTABILISING	-0.5
rs1957777698	T>A	M122K	DECREASE	6	DECREASE	6	DESTABILISING	-1.1
rs2137506152	C>T	R173W	DECREASE	8	DECREASE	8	DESTABILISING	-0.18
rs2500200512	C>A	F119L	DECREASE	6	DECREASE	6	DESTABILISING	-1.17
rs2500200544	G>T	M122I	DECREASE	2	DECREASE	2	DESTABILISING	-1.63
rs2500206228	T>A	F165Y	DECREASE	3	DECREASE	3	DESTABILISING	-0.25
rs2500206438	G>A	E187K	DECREASE	7	DECREASE	7	DESTABILISING	-0.49
rs2500206466	G>C	W193C	DECREASE	7	DECREASE	7	DESTABILISING	-0.0

*a. a mutation: amino acid mutation **RI: Reliability Index.
